# Zeeman-Field-Tuned Topological Phase Transitions in a Two-Dimensional Class-*DIII* Superconductor

**DOI:** 10.1038/srep25503

**Published:** 2016-05-05

**Authors:** W. Y. Deng, H. Geng, W. Luo, L. Sheng, D. Y. Xing

**Affiliations:** 1 National Laboratory of Solid State Microstructures and Department of Physics, Nanjing University, Nanjing 210093, China; 2 Collaborative Innovation Center of Advanced Microstructures, Nanjing University, Nanjing 210093, China

## Abstract

We investigate the topological phase transitions in a two-dimensional time-reversal invariant topological superconductor in the presence of a Zeeman field. Based on the spin Chern number theory, we find that the system exhibits a number of topologically distinct phases with changing the out-of-plane component of the Zeeman field, including a quantum spin Hall-like phase, quantum anomalous Hall-like phases with total Chern number *C* = −2, −1, 1 and 2, and a topologically trivial superconductor phase. The BdG band gap closes at each boundary of the phase transitions. Furthermore, we demonstrate that the zero bias conductance provides clear transport signatures of the different topological phases, which are robust against symmetry-breaking perturbations.

Topological insulators (TIs) have bulk insulating energy gaps and gapless edge or surface states, which are protected by both the bulk band topology and time-reversal (TR) symmetry^1^,^2^,^3^,^4^. The two-dimensional (2D) TIs are also called the quantum spin Hall (QSH) systems, whose topological properties can be described by the *Z*_2_ index^5^,^6^ or spin Chern numbers^7^,^8^. While the *Z*_2_ index and spin Chern numbers yield an equivalent description for TR-invariant systems, the robustness of the spin Chern numbers does not rely on any symmetries^8^,^9^. The spin Chern numbers have been employed to study the TIs in the presence of an exchange field, which breaks the TR symmetry^10^. In such systems, various interesting topological phases can be realized, including the quantum anomalous Hall (QAH) phase^11^, TR-symmetry-broken QSH phase^12^,^13^, Weyl semimetal phase^14^. In the TR-symmetry-broken QSH phase, while the edge states are usually gapped, signaling the presence of backward scattering, an interesting measurable topological spin pumping effect from the bulk can happen, as a direct manifestation of the nontrivial bulk band topology, when time-periodic gate voltage and ac electric field are suitably applied^15^.

Topological superconductors (TSCs) are the superconductor analogue to the TIs, which have gapless Andreev edge states on the boundary^16^,^17^. Because the zero-energy modes of the edge states, known as the Majorana fermions, have potential applications in topological quantum computations^18^,^19^,^20^, TSCs have been attracting much theoretical and experimental attention in recent years^21^,^22^,^23^. The first type TSCs are the class *D* TSCs with broken TR symmetry^24^,^25^. A 2D system of the class *D* TSC is characterized by a nonzero Chern number and chiral gapless edge states^26^,^27^, in analogy to a quantum Hall system. Several systems have been proposed as possible candidates for the class *D* TSCs, including conventional semiconductor wires with Rashba spin-orbit coupling^28^,^29^,^30^,^31^ and the TIs^32^,^33^, when the s-wave superconductivity is induced in the systems through the proximity effect. Some transport signatures of the existence of the class *D* TSCs have been observed experimentally^34^,^35^,^36^,^37^.

Another type of TSCs are the class-*DIII* TSCs with TR symmetry^38^,^39^. Unlike the class *D* TSCs, a 2D system of the TR invariant (TRI) TSC possesses helical gapless edge states, which are protected by the bulk topological invariant and TR symmetry^38^. The topology of the BdG bulk bands can be described by the *Z*_2_ index^39^, similarly to a QSH system. There are some proposals for realizing one-dimensional (1D) and 2D TRI TSCs by utilizing the proximity effect of superconductor, without assuming exotic electron-electron interactions^40^,^41^,^42^,^43^,^44^. Especially, Zhang *et al.* proposed that 1D and 2D TRI TSCs can be realized via the proximity effect between nodeless *s*_±_-wave iron-based superconductors and semiconductors with large Rashba spin-orbit interactions^44^. They also studied the evolution of the Majorana pairs in the 1D model in the presence of a Zeeman field, which leads to different zero-bias conductance (ZBC) peaks, as an experimental signature in tunneling spectroscopy. Interestingly, they found that the Majorana pairs still exist in the presence of a Zeeman field along the *x* or *z* direction, before the first transition occurs. Because the *Z*_2_ index can not be defined in the absence of the TR symmetry, they emphasized that the system is essentially topologically trivial. This conclusion is however arguable, as a transition from a topologically nontrivial phase to a trivial phase is in principle attributable to the change in a topological invariant. The phase diagram of the 2D TRI TSCs in the presence of a Zeeman field has not been investigated. Since the spin Chern numbers are independent of any symmetries, they are more suited to describe the topological phase transitions in such a system.

In this work, we investigate the topological phase transitions in a 2D TRI TSC in the presence of a Zeeman field. By calculation of the spin Chern numbers and BdG edge state spectra, we find that a number of topologically distinct phases can occur with changing the *z* component of the Zeeman field, including a QSH-like phase, QAH-like phases with charge Chern number *C* = −2, −1, 1 and 2, and a trivial superconductor phase. While the charge Chern number vanishes in the QSH-like phase, we reveal that the QSH-like phase is topologically nontrivial in the bulk, characterized by nonzero spin Chern numbers *C*_±_ = ±1 and nontrivial spectral flow of the spin-polarized Wannier functions (SPWFs). In particular, in the QSH-like phase, the helical edge states are gapless with gapped spin spectrum, if the Zeeman field is in the *x* or *z* direction. If the Zeeman field is in the *y* direction, the helical edge states are gapped with gapless spin spectrum. These results conform the general relation between edge states and bulk topological invariant in QSH systems^10^,^45^. We further show that the ZBC can provide clear transport signatures of the different topological phases in experiments, which are robust against symmetry-breaking perturbations.

## Results

### Model Hamiltonian and Topological Phase Transitions

Let us start from the BdG Hamiltonian in the Nambu basis 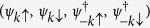, which was used to describe the 2D TSC^44^


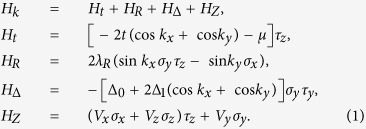


Here, *H*_*t*_, *H*_*R*_, *H*_Δ_, *H*_*Z*_ represent the kinetic energy, Rashba spin-orbit interaction, superconducting pairing potential, and Zeeman field, respectively. *σ*_*i*_ and *τ*_*i*_ with *i* = *x*, *y*, *z* are the Pauli matrices that act on the spin and particle-hole spaces. *t* is the nearest-neighbor hopping amplitude, *μ* is the chemical potential, *λ*_*R*_ is the strength of the Rashba spin-orbit coupling, and Δ_0_ and Δ_1_ are the *s*_±_ wave pairing amplitudes, which can be induced by the proximity effect. In the last term, we include the Zeeman field with components *V*_*i*_, which breaks the TR symmetry. From numerical calculation, we find that the bulk gap remains closed, when the *x* or *y* component of the Zeeman field is increased to be above a certain critical value, without further transitions into other topological phases. Therefore, we will focus on the effect of varying the *z* component of the Zeeman field, in order to study topological phase transitions.

**Figure 1:**
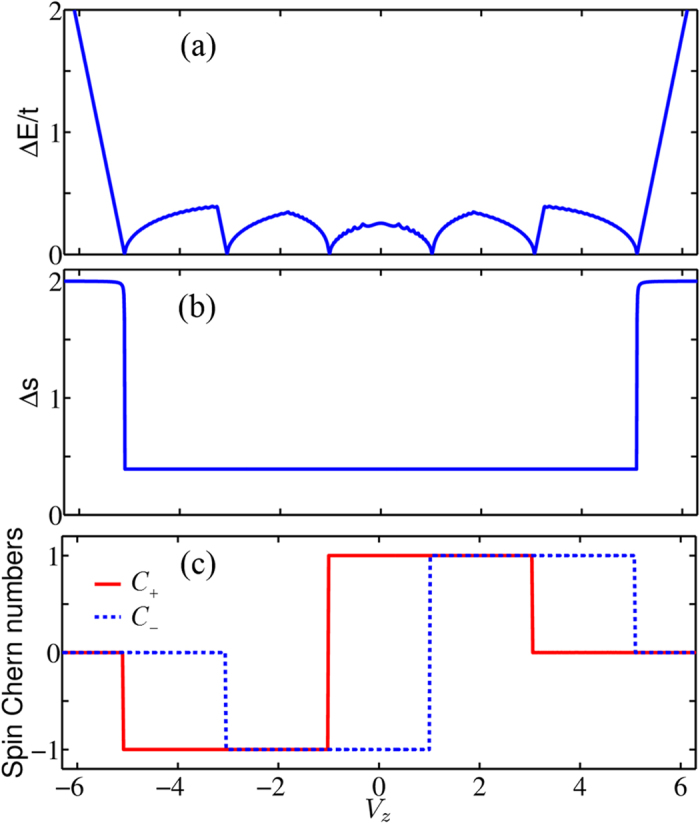
(**a**) BdG band gap Δ*E*, (**b**) spin spectrum gap Δ*s*, and (**c**) spin Chern numbers as functions of the *V*_*z*_ Zeeman field. The parameters are chosen to be *μ* = −1, *λ*_*R*_ = 0.5, Δ_0_ = −0.2, Δ_1_ = 0.2, and *V*_*x*_ = *V*_*y*_ = 0. *t* is taken to be the unit of energy.

The spin Chern numbers for the 2D system can be defined and calculated in a standard way^8^,^9^,^10^, as long as both the BdG band gap and spin spectrum gap stay open. The topological properties of Eq. (1) can be described by the spin Chern numbers associated with the operator 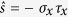 relating to spin. We first consider the case that the initial state without the Zeeman field is topological (the *Z*_2_ index *v* = 1). The BdG band gap Δ*E* between the conduction and valence bands is plotted in Fig. 1(a) as a function of the Zeeman field *V*_*z*_. It is found that with varying *V*_*z*_, the band gap closes six times. The spin spectrum is calculated by diagonalizing the projected spin operator 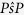, where *P* is the projection operator into the occupied valence bands. The spin spectrum gap Δ*s* is always nonzero, as plotted in Fig. 1(b). We can calculate the spin Chern numbers numerically, and the result is shown in Fig. 1(c). There are six topologically distinct phases characterized by (*C*_+_, *C*_−_) = (1, −1), (1, 1), (−1, −1), (1, 0), (0, 1), and (0, 0), respectively. We see that the points of band gap closing mark the boundaries between different topological phases. The case that the initial state is a trivial phase (*v* = 0) can be studied similarly. In that case, although the phase is trivial with *C*_±_ = 0 for small Zeeman field, topological phases, such as *C*_±_ = 1, can appear with increasing the Zeeman field. For simplicity, in the following, we mainly discuss the properties of the topological phase transitions for positive *V*_*z*_.

**Figure 2:**
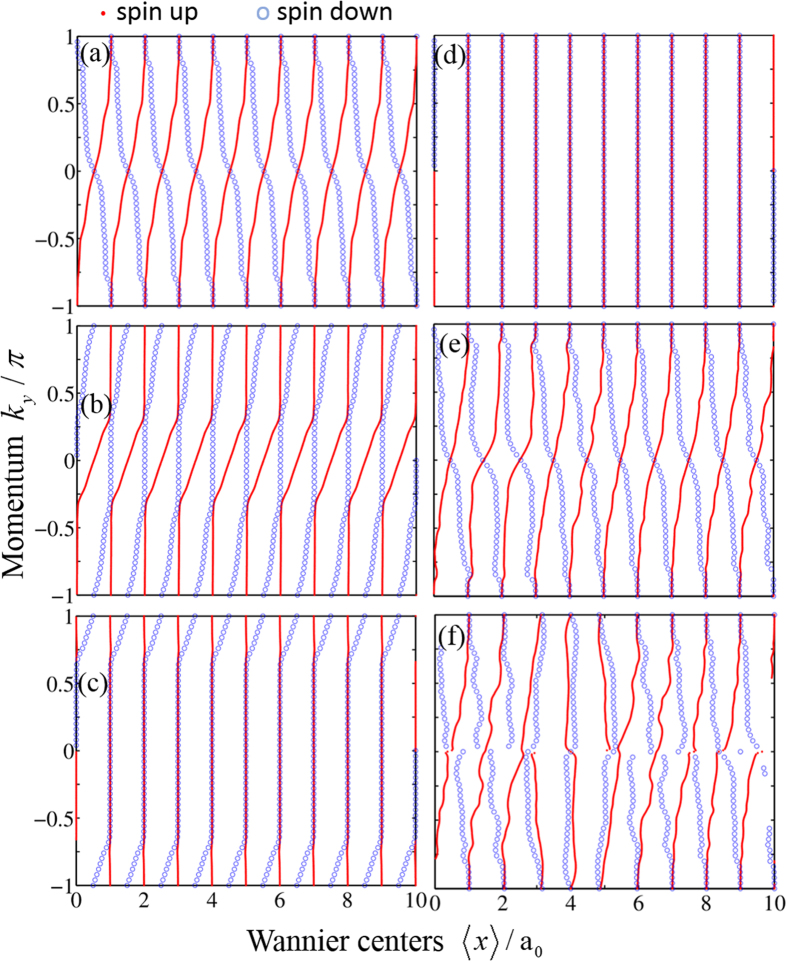
The centers of mass of the SPWFs (horizontal axis) as functions of momentum *k*_*y*_ (vertical) for different values of strength *V*_0_ of magnetic disorder and Zeeman field *V*_*z*_. For clarity, only ten unit cells in the *x* direction with periodic boundary condition are displayed in the figure, where 10% of the atoms are assumed to be replaced by magnetic impurities. a_0_ is the lattice constant. The parameters are taken to be (**a–d**) *V*_0_ = 0 and *V*_*z*_ = 0.5, 2, 4, 5.5, (**e,f**) *V*_0_ = 1.5, 3 and *V*_*z*_ = 0.5. The other parameters are the same as in Fig. 1.

The SPWFs^46^,^47^ are the spin generalization of the conventional Wannier functions, which can also reveal the nontrivial bulk band topology of the system. The centers of mass of the calculated SPWFs are plotted in Fig. 2(a–d) for four different topological phases. In Fig. 2(a), where *V*_*z*_ = 0.5 and *C*_±_ = ±1, all the Wannier centers of the spin-up sector move rightwards, each center shifting on average a lattice constant per cycle (*k*_*y*_ = −*π* → *π*), and those of the spin-down sector move in the opposite direction. According to the general theory^48^, the total displacement of the spin-up (spin-down) Wannier centers per cycle divided by the length of the system is equal to the spin Chern number *C*_+_ (*C*_−_). The movement of the SPWFs shown in Fig. 2(a) is clearly in agreement with the spin Chern numbers. Similarly, the Wannier centers of both spin-up and spin-down sectors move rightwards and shift on average a lattice constant per cycle in Fig. 2(b), which are consistent with *C*_±_ = 1 for *V*_*z*_ = 2. In Fig. 2(c), only the Wannier centers in the spin-down sector move rightwards, being consistent with the spin Chern numbers *C*_+_ = 0 and *C*_−_ = 1 for *V*_*z*_ = 4. In Fig. 2(d), all the SPWFs do not shift in a cycle, corresponding to the topological trivial phase with *C*_±_ = 0 for *V*_*z*_ = 5.5. Therefore, the spectral flows of the SPWFs are fully consistent with the spin Chern numbers.

In order to show the robustness of the nontrivial topological properties described by spin Chern numbers, we assume that magnetic impurities with random positions and classical spins oriented in the *y* direction exist in the system. The Hamiltonian of the magnetic impurities is given by 
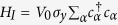
, where *α* runs over all the impurity sites. The magnetic impurities with spins in the *y* direction break both the TR symmetry and BDI symmetry^40^,^44^. The centers of mass of the SPWFs as functions of *k*_*y*_ are obtained numerically for different disorder strengths in the topological phase *C*_±_ = ±1, as shown in Fig. 2(e,f). We can see that in the absence of disorder, *V*_0_ = 0, as shown in Fig. 2(a), all the flowing paths of the Wannier centers of the spin-up or spin-down sector are the same due to the periodicity of the system. In the presence of disorder with strength *V*_0_ = 1.5, as shown in Fig. 2(e), the flowing paths of the Wannier centers have small deformations, but the nontrivial spectral flow remains robust. When the magnetic disorder is strong enough, as shown in Fig. 2(f), the ordered movement of the Wannier centers is eventually interrupted, although some centers still rearrange locally. This means that the system becomes topologically trivial, as spin Chern numbers change to *C*_±_ = 0.

### BdG energy and spin spectra of a ribbon

**Figure 3:**
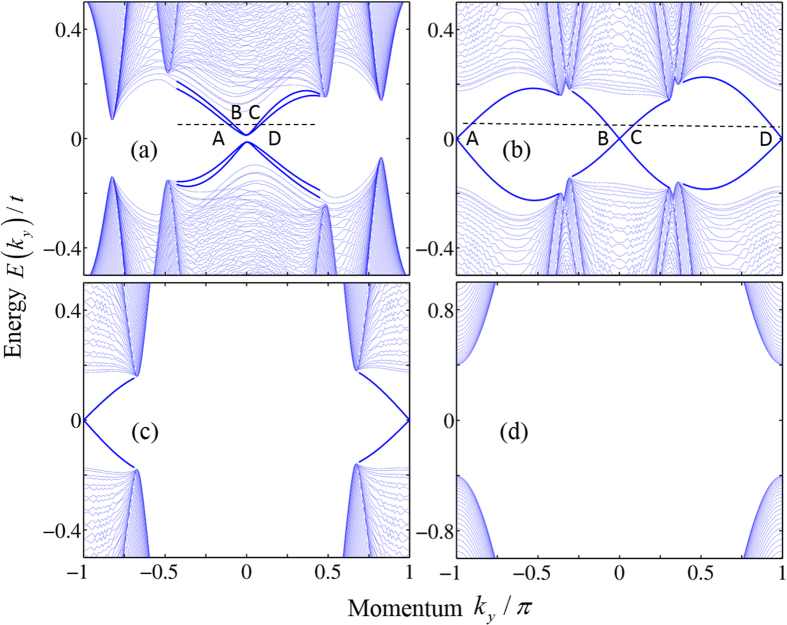
The BdG spectrum of a 2D ribbon running along the *y* direction. The Zeeman field is chosen to be *V*_*x*_ = 0.05, *V*_*y*_ = 0.02, (**a**) *V*_*z*_ = 0.5, (**b**) *V*_*z*_ = 2, (**c**) *V*_*z*_ = 4, and (**d**) *V*_*z*_ = 5.5. The other parameters are the same as in Fig. 1.

To study the edge states in each phase, we calculate the BdG energy spectrum of a 2D ribbon, which runs in the *y* direction and has a width of 100 atoms in the *x* direction. We will set nonzero *V*_*x*_ and *V*_*y*_, but the phase diagram is almost the same as that shown Fig. 1(c), except for small shifts of the phase boundaries. In the *C*_±_ = ±1 phase for *V*_*z*_ = 0.5, there are four edge states in the BdG spectrum, which are labeled as *A*, *B*, *C* and *D* at a given Fermi level, as shown in Fig. 3(a). For nonzero *V*_*x*_ and *V*_*y*_, both the TR and BDI symmetries are broken, and the edge states have a small energy gap. Through the analysis of the spatial distribution and spin polarization of the wave functions, we find that state *A* has spin-up polarization and state *D* has spin-down polarization. They are mainly localized near the right boundary. State *B* is spin-down polarized and state *C* is spin-up polarized, being localized near the left boundary. From the slopes of the energy dispersion curves, it is easy to see that states *A* and *D* are counterpropagating, as well as states *B* and *C*. Therefore, the edge states are helical, and the *C*_±_ = ±1 phase is a QSH-like phase.

Similar analysis can be applied to other phases. In the phase *C*_±_ = 1 for *V*_*z*_ = 2, the BdG energy spectrum is shown in Fig. 3(b). There are also four different edge states labeled as *A*, *B*, *C* and *D* in the band gap. One can find that state *A* is spin-down polarized, and state *C* is spin-up polarized. They are located near the right boundary, and both propagate along the *y* direction. In contrary, the spin-up polarized state *B* and spin-down polarized state *D* are located near the left boundary, and propagate along the −*y* direction. Therefore, the *C*_±_ = 1 is a QAH-like phase with total Chern number *C* ≡ (*C*_+_ + *C*_−_) = 2. In the phase *C*_+_ = 0, *C*_−_ = 1, as shown in Fig. 3(c), only a pair of chiral edge states with spin-down polarization are found in the BdG spectrum, indicating that the system is a QAH-like phase with *C* = 1. The phase *C*_±_ = ±0 is the topologically trivial superconductor phase, as shown in Fig. 3(d), without edge states appearing in the BdG band gap. Therefore, the 2D system undergoes a transition sequence through a QSH-like phase, a *C* = 2 QAH-like phase, a *C* = 1 QAH-like phase and a topologically trivial phase, with increasing the *z* component *V*_*z*_ of the Zeeman field.

**Figure 4:**
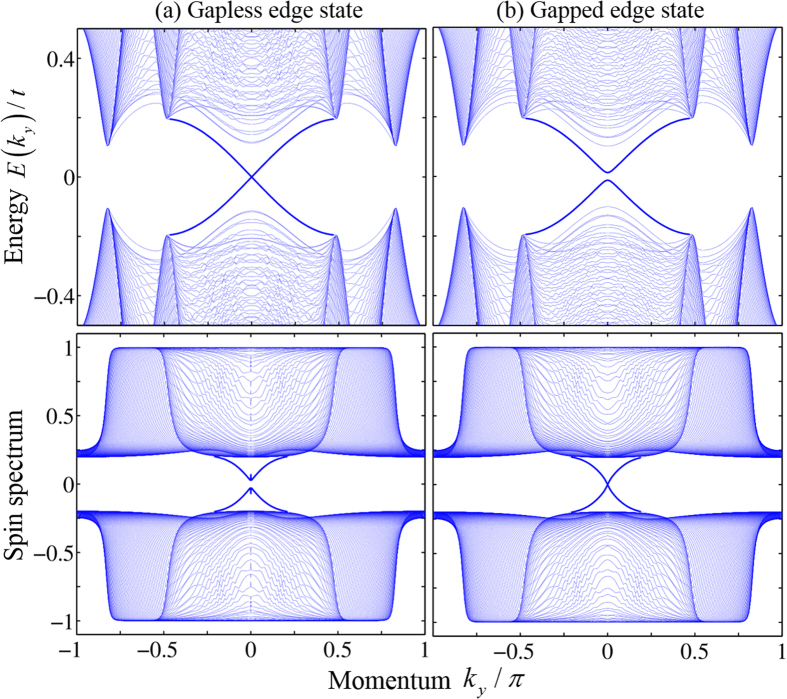
The BdG spectrum (upper panel) and spin spectrum of *PŝP* (lower panel) of a 2D ribbon. The parameters are chosen to be *V*_*x*_ = 0, *V*_*z*_ = 0.5, (**a**) *V*_*y*_ = 0, and (**b**) *V*_*y*_ = 0.02. The other parameters are the same as in Fig. 3.

In a QSH system, the nontrivial band topology guarantees that edge states must appear in the bulk band gap, which could be gapped or gapless in energy spectrum, depending on the symmetries and local microscopic structures near the boundaries^45^. When the energy spectrum is gapped, the spin spectrum must be gapless on the edge. The BdG energy and spin spectra are plotted in Fig. 4. The spin spectrum of 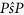 is obtained by diagonalizing the matrix, whose elements are given by 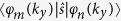 with *m* and *n* running over all the occupied states *φ*(*k*_*y*_). For *V*_*y*_ = 0, due to the BDI symmetry, the edge states are still gapless in the absence of the TR symmetry, as shown in Fig. 4(a), but the spin spectrum is gapped. When the BDI symmetry is broken for *V*_*y*_ = 0.02, the edge states are gapped, but the spin spectrum is gapless, as shown in Fig. 4(b). These numerical results are in consistence with the general theoretical argument that either the energy or spin spectral gap must close on the edge in a QSH phase^45^.

### Zero bias conductance

When the TSC is attached to a normal lead in the 1D TSC system, the ZBC is quantized due to the resonant Andreev reflection^49^,^50^. The ZBC peak is 2*e*^2^/*h* induced by a single Majorana zero mode in class *D* TSCs and 4*e*^2^/*h* induced by a pair degenerate zero modes in class *DIII* TSCs^40^,^44^. Here, we study the ZBC of the 2D system with *k*_*y*_ as a parameter, to find new features in the three topological phases for *V*_*z*_ > 0. We focus on the cases that *k*_*y*_ = 0 and *k*_*y*_ = *π*, where zero Majorana modes may exist.

**Figure 5:**
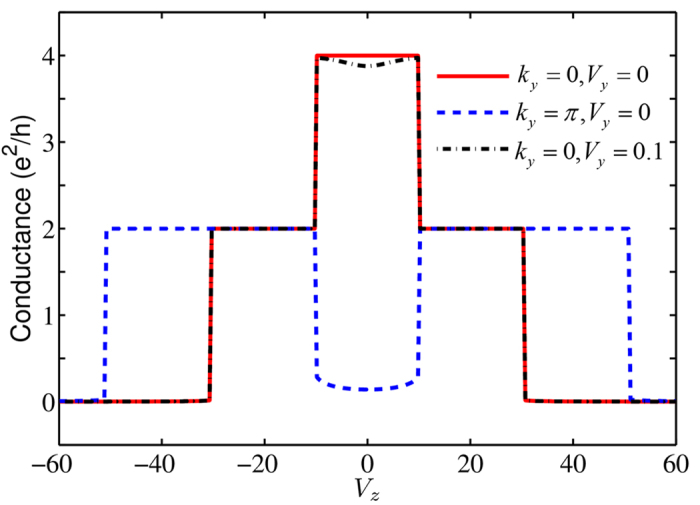
The ZBC for the TSC as a function of the *V*_*z*_ Zeeman field for different values of *k*_*y*_ and *V*_*y*_. The other parameters for the TSC are the same as in Fig. 1. The parameters in the lead are chosen to be *t_*L*_* = *t*, *µ*_*L*_ = *µ* for *k*_*y*_ = 0, and *µ*_*L*_ = –*µ* for *k*_*y*_ = π.

Numerical calculations were performed by using the Kwant package^51^. The ZBCs from the TSC to a normal lead as a function of the *z* component *V*_*z*_ of the Zeeman field are plotted in Fig. 5. For *k*_*y*_ = 0 and *V*_*y*_ = 0, the quantized ZBC peak is 4*e*^2^/*h* in the QSH-like phase, then reduces to 2*e*^2^/*h* in the QAH-like phase, and turns to zero in the insulator phase. These are consistent with the number of zero modes at *k*_*y*_ = 0 in the respective phases. Similarly, for *k*_*y*_ = *π*, there exists a non-degenerate zero mode in the QAH-like phases with *C* = 1 or 2, so the quantized ZBC peak is 2*e*^2^/*h* for these two phases. Especially, in the QSH-like phase, when the BDI symmetry is broken by nonzero *V*_*y*_ of the Zeeman field, the ZBC at *k*_*y*_ = 0 deviates from the quantized value 4*e*^2^/*h* slightly, rather than drops to zero immediately as in a topologically trivial phase. This reflects the fact that the system is still topological, except that weak backscattering is present in the transport of the Majorana fermions.

## Conclusion

In this work, we have studied the topological phase transitions in a 2D TRI TSC in the presence of a Zeeman field, based upon the spin Chern numbers. It is found that with varying the *z* component of the Zeeman field, a number of topologically distinct phases can appear, including a QSH-like phase, QAH-like phases with *C* = ±1 and ±2, and a topologically trivial superconductor phase. The transitions are always accompanied by the BdG band gap closing. It is revealed that the nontrivial topological properties of the bulk wavefunctions remain robust against magnetic disorder. The BdG energy and spin spectra of the edge states calculated for a 2D ribbon are consistent with the topological characterization using the spin Chern numbers. Moreover, the basic characteristics of the ZBC are investigated, which can be used to identify the different topological phases experimentally.

## Additional Information

**How to cite this article**: Deng, W. Y. *et al.* Zeeman-Field-Tuned Topological Phase Transitions in a Two-Dimensional Class-*DIII* Superconductor. *Sci. Rep.* **6**, 25503; doi: 10.1038/srep25503 (2016).
